# A systematic review of the cost-effectiveness of targeted therapies for metastatic non-small cell lung cancer (NSCLC)

**DOI:** 10.1186/1471-2466-14-192

**Published:** 2014-12-04

**Authors:** Ansgar Lange, Anne Prenzler, Martin Frank, Heiko Golpon, Tobias Welte, J-Matthias von der Schulenburg

**Affiliations:** Leibniz University Hannover, Center for Health Economics Research Hannover (CHERH), Otto-Brenner-Str. 1, D-30159 Hannover, Germany; Hannover Medical School, Clinic for Pneumology, Hannover, Germany

**Keywords:** Non-small cell lung cancer, Monoclonal antibody, Bevacizumab, Erlotinib, Gefitinib, Crizotinib, Afatinib, Targeted therapy, Health economics, Cost-effectiveness analysis, Cost-utility analysis, Tyrosine kinase inhibitors

## Abstract

**Background:**

Non-small cell lung cancer (NSCLC) imposes a substantial burden on patients, health care systems and society due to increasing incidence and poor survival rates. In recent years, advances in the treatment of metastatic NSCLC have resulted from the introduction of targeted therapies. However, the application of these new agents increases treatment costs considerably. The objective of this article is to review the economic evidence of targeted therapies in metastatic NSCLC.

**Methods:**

A systematic literature review was conducted to identify cost-effectiveness (CE) as well as cost-utility studies. Medline, Embase, SciSearch, Cochrane, and 9 other databases were searched from 2000 through April 2013 (including update) for full-text publications. The quality of the studies was assessed via the validated Quality of Health Economic Studies (QHES) instrument.

**Results:**

Nineteen studies (including update) involving the MoAb bevacizumab and the Tyrosine-kinase inhibitors erlotinib and gefitinib met all inclusion criteria. The majority of studies analyzed the CE of first-line maintenance and second-line treatment with erlotinib. Five studies dealt with bevacizumab in first-line regimes. Gefitinib and pharmacogenomic profiling were each covered by only two studies. Furthermore, the available evidence was of only fair quality.

**Conclusion:**

First-line maintenance treatment with erlotinib compared to Best Supportive Care (BSC) can be considered cost-effective. In comparison to docetaxel, erlotinib is likely to be cost-effective in subsequent treatment regimens as well. The insights for bevacizumab are miscellaneous. There are findings that gefitinib is cost-effective in first- and second-line treatment, however, based on only two studies. The role of pharmacogenomic testing needs to be evaluated. Therefore, future research should improve the available evidence and consider pharmacogenomic profiling as specified by the European Medicines Agency. Upcoming agents like crizotinib and afatinib need to be analyzed as well.

**Electronic supplementary material:**

The online version of this article (doi:10.1186/1471-2466-14-192) contains supplementary material, which is available to authorized users.

## Background

Lung cancer is one of the most common cancers in the world and accounts for 12.7% of all new cancers in 2008 [1]. The high world incidence of lung cancer is expected to increase in the next decades, particularly in countries with medium standards due to adoption of unhealthy western lifestyles such as smoking [2].

Non-small cell lung cancer (NSCLC) accounts for about 9 out of 10 cases of all lung cancers [3]. The survival rate for patients with NSCLC is markedly influenced by the stage at diagnosis [4]. At initial diagnosis approximately 25% of patients have regional metastasis and 55% of patients have already developed distant metastasis due to the high vascularization and rich supply of lymphatic vessels of the lung [5]. This is a reason, why lung cancer is considered the most common cause of death from cancer (18.2% of all cancer related deaths) [1].

Surgery in combination with adjuvant chemotherapy is a potentially curative option in early-stage disease. If patients are not eligible for surgery, radiation therapy combined with chemotherapy is a treatment alternative. In patients with metastatic disease, platinum-based chemotherapy with carboplatin or cisplatin has been considered the main treatment option for decades [6]. However, survival rates for lung cancer patients, especially when they have developed metastasis, are quite poor.

More recently, advances in the treatment of NSCLC have resulted from the addition of targeted anti-cancer drugs to chemotherapy. These targeting agents aim to inhibit the tumor growth by interfering with specific proteins (cell signaling) involved in tumor progression, e.g. by blocking the signal transduction through Epidermal Growth Factor Receptor (EGFR), Vascular Endothelial Growth Factor (VEGF) or Anaplastic Lymphoma Kinase (ALK) gene.

Currently approved targeted agents for the treatment of advanced NSCLC are the VEGF antibody bevacizumab; erlotinib and gefitinib (all targeting EGFR) as well as crizotinib targeting ALK. Another EGFR tyrosine kinase inhibitors, Afatinib, is currently under review at the European Medicine Agency (EMA) and US Food and Drug Administration (FDA). Originally, Merck KGaA sought to get approval by EMA for its EGFR antibody cetuximab for the treatment of NSCLC. However, in September 2012, the company withdrew its application [7]. An overview of the targeted agents for the treatment of metastatic NSCLC and the current FDA and EMA approval status is provided in Table 1.

**Table 1:** **Targeted agents for the treatment of metastatic NSCLC**

Generic drug name	Target	Approved population	Type	EMA approved treatment regimens in NSCLC	FDA approved treatment regimens in NSCLC
Afatinib	EGFR	EGFR positive NSCLC patients	Tyrosine-kinase inhibitor	*Under review*	*Under review*
Bevacizumab	VEGF	All NSCLC patients	Recombinant humanized monoclonal antibody	In addition to platinum-based chemotherapy for first-line treatment of adult patients with unresectable advanced, metastatic or recurrent NSCLC other than predominantly squamous cell histology (Aug 2007)	Non-squamous NSCLC, with carboplatin and paclitaxel for first line treatment of unresectable, locally advanced recurrent or metastatic disease (Oct 2006)
Cetuximab	EGFR		Chimeric monoclonal IgG_1_ antibody	*None*; *Merck KGaA withdrew its application formally in Sep 2012*	-
Crizotinib	ALK	ALK positive NSCLC patients	Anaplastic lymphona kinase inhibitor	Adult patients with previously treated ALK-positive NSCLC (Oct 2012)	Patients with locally advanced or metastatic NSCLC that is ALK-positive as detected by an FDA-approved test (Aug 2011)
Erlotinib	EGRF	EGFR positive NSCLC patients	Tyrosine-kinase inhibitor	Patients with locally advanced or metastatic NSCLC after failure of at least one prior chemotherapy regimen (Oct 2005)	• Treatment of patients with locally advanced or metastatic NSCLC after failure of at least one prior chemotherapy regimen (Nov 2004)
					• Maintenance treatment of patients with locally advanced or metastatic NSCLC whose disease has not progressed after four cycles of platinum-based first-line chemotherapy (April 2010)
					• First-line treatment of metastatic NSCLC patients whose tumors have epidermal growth factor receptor (EGFR) exon 19 deletions or exon 21 (L858R) substitution mutations (May 2013)
					Monotherapy for the continued treatment of patients with locally advanced or metastatic NSCLC after failure of both platinum-based and docetaxel chemotherapies who are benefiting or have benefited (May 2003). The approval was limited to cancer patients who, in the opinion of their treating physician, are currently benefiting, or have previously benefited, from gefitinib treatment (Jun 2005)
Gefitinib	EGFR	EGFR positive NSCLC patients	Tyrosine-kinase inhibitor	Adult patients with locally advanced or metastatic NSCLC with activating mutations of EGFR (Jun 2009)	

(Status as of May 2013).

Despite the potential benefit of targeted agents in the treatment of NSCLC, their application is discussed controversial due to their high prices [8]. Hence, it is necessary to assess the economic impact of the use of these agents in NSCLC. Moreover, health economic evaluations are necessary to support price negotiations as well as reimbursement decisions.

The objective of this article is therefore to review and assess the economic evidence of treatments with targeted agents in advanced NSCLC. A systematic literature review was conducted to identify and analyze cost-effectiveness analysis (CEA) and cost-utility analysis (CUA) studies that used modelling approaches. The quality of the studies was assessed via a validated assessment tool.

## Method

Prior to the systematic literature research, PICO (Population – Indication – Comparator – Outcome) elements were defined according to the objective of this review (see Table 2).

**Table 2:** **Review objective and PICO elements**

**Review objective**	The objective of this article is to review the economic evidence of treatment of NSCLC with targeted agents.
**Participants**	Studies of participants diagnosed with NSCLC. Studies were not restricted based on age of the participants or treatment lines.
**Interventions/Comparison**	Studies about treatments with approved targeted agents or agents still going through the approval process. The review is not limited to specific comparators
**Outcomes**	ICER, e.g. cost per QALY or cost per life year gained/saved

A systematic literature search in AMED, BIOSIS Previews, Cochrane Central Register of Controlled Trials, Cochrane Database of Systematic Reviews, DAHTA-Datenbank, Database of Abstracts of Reviews of Effects, EMBASE, EMBASE Alert, Health Technology Assessment Database, MEDLINE, NHS Economic Evaluation Database, SciSearch and SOMED database was conducted in September 2012 using the meta-database of the German Institute for Medical Documentation and Information (DIMDI). The search process was repeated again in April 2013 in order to keep the review up to date. The full-text search included publications published in English and German during 2000 to 2012. The following search terms (English and German) were used and finally combined with AND: (i) ({non small cell lung OR non small cell bronchial OR non small lung OR non small bronchial OR nsclc} AND {cancer OR carcinom? OR tumour OR neoplasm?}); (ii) (stadium IIIb OR stadium IV OR stadium 3b OR stadium 4 OR stage IIIb OR stage Iv OR stage 3b OR stage 4 OR metasta? OR advanced); (iii) (cetuximab OR gefitinib OR bevacizumab OR erlotinib OR crizotinib OR afatinib); (iv) (Cost OR Cost? OR efficien?). The “?” was used as a wild card to represent any number of characters. In addition, a hand search was conducted. (*Note*: *The search algorithm reveals that we included the EGFR*-*antibody cetuximab in our search terms. This is due to the fact that the withdrawal of Merck KGaA occurred after the first data extraction in September 2012. Nevertheless*, *we did not consider cetuximab in the final assessment*).

**Figure 1:**
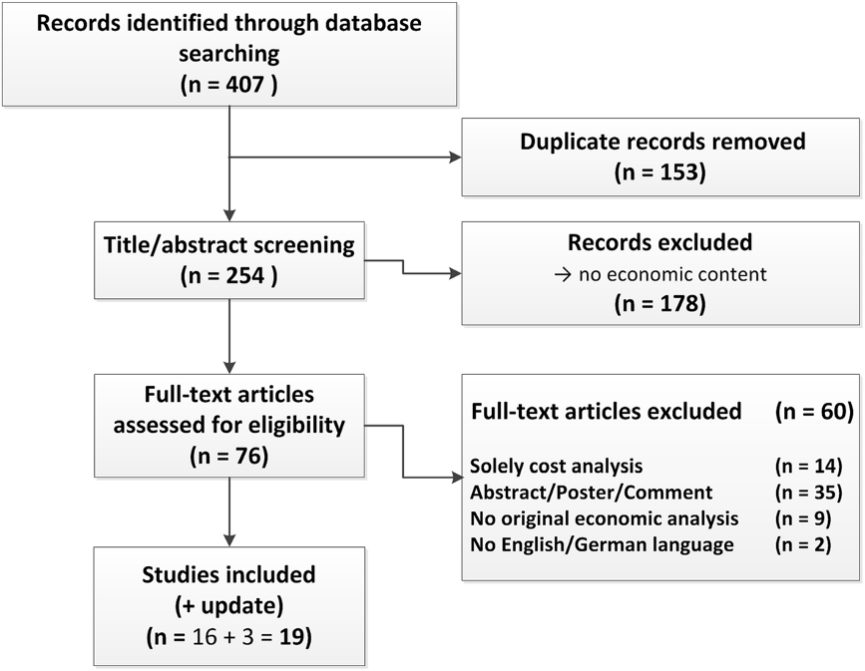
**Flow diagram of articles identified and evaluated based on inclusion criteria.**

Titles and abstracts of all identified publications were reviewed independently by two researchers. Only original studies published in a full text were included. The eligibility of the studies for the review was also assessed independently. Disagreements were settled through discussion. Figure 1 summarizes the search process.

We collected data for each included article across a range of elements, including e.g. authors, journal, study question, population, intervention, setting, perspective and, funding source (see Additional file 1).

Results in terms of incremental costs, cost-effectiveness ratios and cost-utility ratios were converted to US dollars at the exchange rate prevalent in the year of publication to ensure comparability [9].

The Quality of Health Economic Studies (QHES) instrument was used to evaluate the quality of the included studies (Table 3) [10]. QHES is a validated instrument designed to measure the quality of health economic analyses. Each study was scored in 16 items for quality between 0 and 100, whereby 0 represents lowest quality and 100 highest quality. Each item has a weighted point value that was generated from regression analysis. No partial points per item are intended [10]. After summing-up points for the 16 items the studies are grouped by the following quartiles: (1) extremely poor quality (0–24); (2) poor quality (25–49); (3) fair quality (50–74); and (4) high quality (75–100) [11].

**Table 3:** **The QHES instrument**

	Questions	Points	Yes	No
**1**.	Was the study objective presented in a clear, specific, and measurable manner?	**7**		
**2**.	Were the perspective of the analysis (societal, third-party payer, etc.) and reasons for its selection stated?	**4**		
**3**.	Were variable estimates used in the analysis from the best available source (i.e., randomized control trial - best, expert opinion - worst)?	**8**		
**4**.	If estimates came from a subgroup analysis, were the groups prespecified at the beginning of the study?	**1**		
**5**.	Was uncertainty handled by (1) statistical analysis to address random events, (2) sensitivity analysis to cover a range of assumptions?	**9**		
**6**.	Was incremental analysis performed between alternatives for resources and costs?	**6**		
**7**.	Was the methodology for data abstraction (including the value of health states and other benefits) stated?	**5**		
**8**.	Did the analytic horizon allow time for all relevant and important outcomes? Were benefits and costs that went beyond 1 year discounted (3% to 5%) and justification given for the discount rate?	**7**		
**9**.	Was the measurement of costs appropriate and the methodology for the estimation of quantities and unit costs clearly described?	**8**		
**10**.	Were the primary outcome measure(s) for the economic evaluation clearly stated and did they include the major short-term, long-term, and negative outcomes?	**6**		
**11**.	Were the health outcomes measures/scales valid and reliable? If previously tested valid and reliable measures were not available, was justification given for the measures/scales used?	**7**		
**12**.	Were the economic model (including structure), study methods and analysis, and the components of the numerator and denominator displayed in a clear, transparent manner?	**8**		
**13**.	Were the choice of economic model, main assumptions, and limitations of the study stated and justified?	**7**		
**14**.	Did the author(s) explicitly discuss direction and magnitude of potential biases?	**6**		
**15**.	Were the conclusions/recommendations of the study justified and based on the study results?	**8**		
**16**.	Was there a statement disclosing the source of funding for the study?	**3**		
Total Points		**100**		

The QHES evaluation was conducted independently by two researchers. Question 3 was interpreted as meaning that a justification for the choice of data must be given to fulfill this criteria. Therefore, using data from randomized controlled trials (RCT’s) without any justification of choice, e.g. by a systematic literature review, was categorized as not sufficient. The price years for the measurement of costs were not stated occasionally and in case associated with item 9.

## Results

The database search identified 407 records, 153 of them were duplicates. After titles and/or abstract screening of the remaining 254 records, 178 publications were excluded because they did not cover any relevant economic content. 76 full text articles were assessed for eligibility. Inclusion criteria were fulfilled by 16 articles (Figure 1). Three additional records were identified by a subsequent database search in April 2013.

All in all, 19 studies were included in the assessment. All publications vary regarding a plurality of elements, particularly country setting, treatment combinations and treatment line. Table 4 provides a detailed summary of the results of the identified CEA und CUA.

**Table 4:** **Summary of the included publications**

Author (Publication year)	Country/perspective (Pharma sponsored?)	Treatment line	Treatment	Incremental costs	LYG gained	QALY’s gained	ICER (per LYG)	ICER (per QALY)
				In US-$			In US-$	In US-$
			**Erlotinib vs. BSC/chemotherapy**					
Wang et al. (2013) [16]	China/health care system (no)	First	Erlotinib vs. carboplatin-gemcitabine chemotherapy	$ 48,119***	0.84	0.58	$ 30,455	$ 85,927
Vergnenegre et al. (2012) [13]	France/payer (yes)	First maintenance	Erlotinib plus BSC vs. BSC	11,140 € ($ 15,476*)	0.28		39,783 € ($ 55,266*)	
	Germany/payer (yes)			13,141 € ($ 18,255*)	0.28		46,931 € ($ 65,196*)	
	Italy/payer (yes)			7,808 € ($ 10,847*)	0.28		27,885 € ($ 38,738*)	
Walleser et al. (2012) [14]	UK/payer (no)	First maintenance	Erlotinib vs. BSC	7,898 € ($ 10,460*)	0.39		20,711 € ($ 27,430*)	
	Germany/payer (no)			9,580 € ($ 12,688*)			25,124 € ($ 33,275*)	
	France/payer (no)			8,873 € ($ 11,752*)			23,271 € ($ 30,821*)	
	Spain/payer (no)			8,488 € ($ 11,242*)			22,261 € ($ 29,483*)	
	Italy/payer (no)			8,149 € ($ 10,793*)			21,368 € ($ 28,300*)	
Klein et al. (2010) [15]	USA/payer (yes)	First maintenance	Erlotinib vs. pemetrexed	$ -24,474	-0.1629		no statement	
			Erlotinib vs. BSC	$ 7,470	0.0982		$ 76,069**	
Araújo et al. (2008) [17]	Portugal/health care system (yes)	Subsequent	Erlotinib vs. BSC	10,366 € ($ 15,184)	0.15	0.064	70,424 € ($ 103,159)	161,742 € ($ 236,924)
			Erlotinib vs. docetaxel	-2,784 € ($ -4,078)	0	0.025	Dominant	Dominant
			Erlotinib vs. pemetrexed	-6,284 € ($ -9,205)	0	0.009	Dominant	Dominant
Carlson et al. (2008) [12]	USA/payer (yes)	Subsequent	Erlotinib vs. docetaxel	$ -2,127		0.01		Dominant
			Erlotinib vs. pemetrexed	$ -6,782		0.01		Dominant
Lewis et al. (2010) [18]	UK/NHS (yes)	Subsequent (second)	Erlotinib vs. docetaxel	£ -226 ($ -352)		0.032		Dominant
Thongprasert et al. (2012) [19]	Thailand/payer (yes)	Subsequent	Erlotinib vs. docetaxel	$ 1,746		0.0140		$ 124,703
Cromwell et al. (2011) [20]	Canada/health care system (no)	Subsequent	Erlotinib vs. docetaxel	2,891 CAD ($ 2,529)	0.003		Not calculated, no statistical differences	
Cromwell et al. (2012) [21]	Canada/health care system (no)	Subsequent	Erlotinib vs. BSC	11,102 CAD ($ 9,712)	0.25		36,838 CAD ($ 32,226)	
Bradbury et al. (2010) [22]	Canada/health care system (no)	Subsequent	Erlotinib vs. BSC	12,303 CAD ($ 11,454)	0.13		94,638 CAD ($ 88,109)	
			**Gefitinib vs. chemotherapy**					
Zhu et al. (2013) [23]	China/health care system (no)	First	Gefitinib (WT patients only) vs. routine care	$ 26,150	0.74	0.46	$ 35,337	$ 57,066
Thongprasert et al. (2012) [19]	Thailand/payer (yes)	Subsequent	Gefitinib vs. docetaxel	$ -247	/	0.0140	/	Dominant
			**Erlotinib (various combinations)**					
Chouaid et al. (2012) [24]	France/payer (yes)	First	Erlotinib followed by docetaxel and gemcitabine (DG) vs. DG followed by erlotinib (*fit elderly patients*)	3,954 € ($ 5,497)	/	-0.01	/	395,400 € ($ 549,700)
Chouaid et al. (2013) [25]	France/payer (yes)	First	Erlotinib followed by gemcitabine vs. gemcitabine followed by erlotinib (*frail elderly patients*)	130€ ($ 181)	/	-0.02	/	/
Carlson et al. (2009) [26]	USA/societal (no)	Subsequent	EGFR protein expression test (erlotinib if high expression/docetaxel if low expression) vs. No testing (erlotinib monotherapy)	$ 6,274	/	0.04	/	$ 179,612
			EGFR gene copy number test (erlotinib if high number/docetaxel if low number) vs. No testing (erlotinib monotherapy)	$ 9,209	0.12	0.06	$ 78,367	$ 162,018
			**Bevacizumab (plus chemotherapy) vs. chemotherapy**					
Giuliani et al. (2010) [27]	Italy/payer (yes)	First	Bevacizumab plus cisplatin and gemcitabine vs. pemetrexed plus cisplatin	4,007 € ($ 5,566)	0.12		34,919 € ($ 48,509)	
Ahn et al. (2011) [28]	Korea/payer (yes)	First	Bevacizumab plus cisplatin and gemcitabine vs. cisplatin plus pemetrexed	$ 33,322	1.10		$ 30,318	
	Taiwan/payer (yes)	First	Bevacizumab plus cisplatin and gemcitabine vs. cisplatin plus pemetrexed	$ 64,541	1.19		$ 54,317	
Goulart et al. (2011) [29]	USA/payer (no)	First	Bevacizumab plus carboplatin-paclitaxel vs. carboplatin-paclitaxel	$ 71,620	0.23	0.13	$ 308,982	$ 559,610
Klein et al. (2009) [30]	USA/payer (yes)	First	Carboplatin/paclitaxel/bevacizumab vs. cisplatin/pemetrexed	$ 24,528	0.0727	0.0244	$ 337,179	$ 1,006,065
Klein et al. (2010) [15]	USA/payer (yes)	First	Bevacizumab vs. pemetrexed	$ 9,187	-0.0480		Dominated	

*Abbreviations:* *BSC* best supportive care, *CAD* Canadian dollar, *ICER* incremental cost-effectiveness ratio, *LYG* life-year gained, *QALY* quality-adjusted life year, *UK* United Kingdom, *vs.* versus, *WT* wild type.

*Price year assumed **Not calculated by the authors ***Stated by the authors. The components of nominator and denominator, however, indicate that erlotinib is dominant.

### Erlotinib vs. BSC or chemotherapy

Eleven studies analyzed the cost-effectiveness of the treatment with erlotinib compared to BSC or chemotherapy [12–22]: One study focused on first-line treatment [16], three on first-line maintenance treatment [13–15] and seven studies on subsequent treatment lines [12, 17–22]. Wang et al. [16] analyzed the cost-effectiveness of first-line erlotinib monotherapy compared to carboplatin-gemcitabine combination therapy in patients with advanced EGFR mutation-positive NSCLC. They conclude that erlotinib is cost-effective from the perspective of the Chinese health care system. However, their ICER calculation is based on differences in progression free survival (PFS). Vergnenegre et al. [13] estimated the cost-effectiveness of first-line maintenance treatment with erlotinib vs. BSC in a German, France and Italian setting. The authors conclude that erlotinib is cost-effective in first-line maintenance therapy at a time horizon of five years. Restricting patients to those with EGFR wild type, Walleser et al. [14] also assessed the cost-effectiveness of first-line maintenance treatment with erlotinib over a lifetime horizon. They deduce cost-effectiveness of erlotinib for the country settings of UK, Germany, France and Italy. Klein et al. [15] focused on the cost-effectiveness of first-line maintenance therapy with pemetrexed from a US payer perspective; however, the authors have also considered erlotinib in their calculations. They showed that the treatment with erlotinib causes lower costs and lower efficacy when compared to pemetrexed at a time horizon of three years. These results applied to patients with nonsquamous and patients with squamous and nonsquamous histology. Therefore, no ICER was calculated. Furthermore, the reported values indicate that erlotinib is hardly cost-effective when compared to BSC in patients with metastatic nonsquamous NSCLC.

Araújo et al. [17] analyzed erlotinib in subsequent treatment lines versus docetaxel, pemetrexed, and BSC for the Portuguese health care system. They conclude that erlotinib is dominant with lower cost and higher efficacy than docetaxel as well as pemetrexed. However, the ICER per QALY compared to BSC was $ 236,924. Carlson et al. [12] drew a similar conclusion concerning erlotinib vs. docetaxel or pemetrexed for a US setting. Erlotinib seems to be dominant, since more QALYs were gained at lower costs. The cost-effectiveness of erlotinib vs. docetaxel was also analyzed for the setting of the National Health Service (NHS). Lewis et al. [18] concluded that erlotinib is dominant and cost-effective. In contrast, Thongprasert et al. [19] and Cromwell et al. [20] did not reveal cost-effectiveness for erlotinib vs. docetaxel for the Thai and Canadian setting, respectively. Two years costs were higher with only slightly better efficacy for erlotinib in the second-line treatment of advanced NSCLC patients from a Thai payer perspective [19]. However, Cromwell et al. [20] did not discover statistical difference in terms of costs and overall survival (OS) for the second-line treatment with erlotinib. The cost-effectiveness of erlotinib vs. BSC was assessed somewhat opposing in the studies by Cromwell et al. [21] and Bradbury et al. [22] for the Canadian setting. Cromwell et al. [21] revealed a potential cost-effectiveness of erlotinib for the third-line treatment compared to BSC. By contrast, Bradbury et al. [22] reported no cost-effectiveness of erlotinib for second and third-line treatment due to a substantial lower benefit in terms of OS. However, they calculated an increasing effectiveness of erlotinib when the analysis was restricted to a second-line treatment setting.

### Gefitinib vs. chemotherapy

Only two studies were identified that evaluated the cost-effectiveness of gefitinib [19, 23]. Zhu et al. [23] considered EGFR mutation status in their cost-effectiveness model for first-line gefitinib maintenance therapy. Only patients with EGFR mutation-positive NSCLC received gefitinib. They concluded that gene-guided gefitinib maintenance treatment is indicated as a cost-effective option compared to routine follow-up in China. From a Thai payer perspective, Thongprasert et al. [19] revealed that gefitinib is dominant compared to docetaxel in the second-line treatment without taking pharmacogenomic profiling into consideration.

### Erlotinib (various combinations)

Three other studies addressed the cost-effectiveness of treatments including erlotinib [24–26]. The objective of a study by Chouaid et al. [24] was to assess the cost-effectiveness of erlotinib followed by chemotherapy after disease progression, compared to the reverse strategy from the perspective of the French health care system. Chemotherapy included docetaxel and gemcitabine. They focused on a highly specific population of fit elderly; no significant differences in patient outcomes were identified. However, first-line treatment with chemotherapy was slightly more expensive. Chouaid et al. [25] replicated this study design for frail elderly patients and, once again, could not detect meaningful differences in terms of cost-effectiveness. Carlson et al. [26] evaluated the cost-utility of implementing epidermal growth factor receptor (EGFR) testing before initiating second-line therapy with erlotinib. Two testing strategies were compared. The EGFR protein expression test and the EGFR gene copy number test. Within the testing strategies, erlotinib was given to patients with high expression or a high copy number and docetaxel to those with low expression or a low number, respectively. Erlotinib monotherapy, without testing, was used as the comparator. The analysis showed that EGFR testing has the potential to improve quality-adjusted life expectancy in NSCLC. However, the improvement could only be achieved at high costs and the results had a high uncertainty.

### Bevacizumab (plus chemotherapy) vs. chemotherapy

Five studies evaluated the cost-effectiveness of bevacizumab in the first-line treatment of advanced non-squamous NSCLC [15, 27–30]. Four of these studies compared bevacizumab plus chemotherapies to chemotherapies alone [27–30]. Giuliani et al. [27] analyzed bevacizumab in combination with cisplatin and gemcitabine vs. pemetrexed in combination with cisplatin. They conclude that the bevacizumab-based therapy can be considered as cost-effective in Italy. Comparing the same treatment regimens for the Korean and Taiwanese setting, Ahn et al. [28] revealed similar results. Both, Ahn et al. [28] as well as Giuliani et al. [27], used an indirect comparison to obtain efficacy data since no head-to-head trials existed. However, the results of both studies were completely different in terms of LYG. Goulart et al. [29] assessed the cost-effectiveness of bevacizumab plus chemotherapy compared to chemotherapy alone for the US setting. Chemotherapeutic agents were carboplatin and paclitaxel. They concluded that bevacizumab is not cost-effective when added to chemotherapy; neither when considering LYG nor when taking QALY’s into account. The cost-effectiveness of bevacizumab added to carboplatin and paclitaxel in comparison to pemetrexed plus cisplatin was further analyzed by Klein et al. [30]. Marginal benefits in terms of LYG and QALY’s can only be achieved at very high costs. Hence, bevacizumab based treatment is supposed to be not cost-effective. Beyond, Klein et al. [15] concluded that bevacizumab monotherapy is dominated by pemetrexed in the first-line maintenance treatment due to lower efficacy and higher costs.

### Quality assessment (QHES)

The results of the quality assessment using the QHES instrument are presented in Table 5. The table shows how often each criterion was met by the 19 studies. The quality of the included studies is at a fair level (Mean QHES Score: 66.5 SD: 17.2). More than 40% of the studies are classified as high quality and almost half of the studies are of fair quality. Only two studies are classified as poor quality [24, 25].

**Table 5:** **Results of the QHES assessment**

Study	1	2	3	4	5	6	7	8	9	10	11	12	13	14	15	16	Score
Wang et al. (2013) [16]	√	√	x	√	√	√	√	√	√	x	x	√	√	x	x	√	65
Vergnenegre et al. (2012) [13]	√	√	x	√	x	√	x	√	x	x	√	√	x	x	√	√	51
Walleser et al. (2012) [14]	√	√	x	√	√	√	√	√	x	√	x	√	x	x	√	x	61
Klein et al. (2010) [15]	√	√	x	x	x	x	x	√	x	√	√	√	√	x	√	√	57
Araújo et al. (2008) [17]	√	√	x	√	√	√	√	√	x	√	√	x	√	x	x	√	62
Carlson et al. (2008) [12]	√	√	x	√	√	√	√	√	√	√	√	√	√	√	√	√	92
Lewis et al. (2010) [18]	√	√	x	√	x	√	√	√	x	√	√	x	√	x	√	√	61
Thongprasert et al. (2012) [19]	√	√	x	√	x	√	x	√	x	√	√	√	√	x	√	x	61
Cromwell et al. (2011) [20]*	√	√	√	√	x	√	√	√	√	√	√	n.a.	n.a.	√	√	√	76
Cromwell et al. (2012) [21]*	√	√	√	√	x	√	√	√	√	√	√	n.a.	n.a.	√	√	√	76
Bradbury et al. (2010) [22]*	√	√	√	√	√	√	√	√	√	x	√	n.a.	n.a.	√	√	√	79
Zhu et al. (2013) [23]	√	√	x	√	√	√	x	√	√	√	√	√	x	√	√	√	80
Chouaid et al. (2012) [24]	√	√	x	√	x	√	x	x	x	x	√	x	√	x	√	√	43
Chouaid et al. (2013) [25]	√	√	x	√	x	√	x	x	√	x	√	x	x	x	√	√	44
Carlson et al. (2009) [26]	√	√	x	√	√	√	√	√	x	x	√	√	√	√	√	√	78
Giuliani et al. (2010) [27]	√	√	x	√	x	√	x	√	x	√	√	√	√	x	√	√	64
Ahn et al. (2011) [28]	√	√	x	√	√	√	√	√	x	√	√	√	√	x	√	√	78
Goulart et al. (2011) [29]	√	√	x	√	√	√	√	√	√	x	√	√	√	√	√	√	86
Klein et al. (2009) [30]	√	√	x	√	x	x	x	x	x	√	√	√	√	√	√	√	57
**Statement frequency**	19	19	3	18	9	17	11	16	8	12	17	12	12	8	17	17	

*No model is used in the study. Question 12 & 13 are therefore not applicable.

The study objectives as well as the perspective of the analysis are clearly presented by all studies (Question 1 & 2). Only three studies [20–22] justify the data selection e.g. through a systematic literature review. The remaining studies may not have used data from the best available source (Question 3). As mentioned before, using data from RCT without any justification was not considered appropriate. Klein et al. [15] used data from a subgroup analysis, which, however, the authors do not describe properly (Question 4). Uncertainty is not handled sufficiently by more than half of the studies (Question 5) [13, 15, 18–21, 24, 25, 27, 30]. Conducting probabilistic sensitivity analysis usually fulfilled this criterion. All studies perform an incremental analysis, since this was an inclusion criterion for this review. However, Klein et al. [15, 30] do not undertake a full incremental analysis between all available comparators (Question 6). Detailed information on the methods used to derive data or parts of the data is not reported in eight studies (Questions 7) [13, 15, 19, 23–25, 27, 30]. All studies handle time horizons and discounting correctly, except for Klein et al. [30] who do not discount in the base case as well as Chouaid et al. [24, 25] who do not discount nor clarify the time horizon of their model (Question 8). The costs were not measured appropriately in two thirds of the publications (Question 9) concerning the statement of the price year [13, 14, 28, 30], inclusion of all relevant costs [13, 24, 27], description of the estimation of quantities [15, 26, 30], and details on expert panels [17–19]. In seven studies it remains unclear if all long term costs were included (Question 10), due to no consideration of subsequent treatment costs [13, 16, 22, 29], overall insufficient reporting [24, 25], and incomplete cost consideration of the societal perspective [26]. The results by Walleser et al. [14] might be sensitive to the usage of a QoL measurement, whereas Wang et al. [16] have given no justification for using an unusual health outcome measure (Question 11). While three studies [16, 20–22] use no economic model, the model is displayed in an intransparent manner by three studies [18, 24, 25] and the analysis reported selectively by one study [17] (Question 12). No justification for the choice of the model was given by four studies [13, 14, 23, 25] (Question 13). The direction and magnitude of potential bias is not discussed by eleven studies [13–19, 24, 25, 27, 28] (Question 14). With one exception, the conclusions stated by the authors are based on the respective study results and appear to be reasonable. Only Araújo et al. [17] and Wang et al. [16] seem to have selectively reported the results of their models (Question 15). Finally, Walleser et al. [14] as well as Thongprasert et al. [19] do not disclosure the source of funding for the study (Question 16).

## Discussion

This is a comprehensive review that analyses the cost-effectiveness of targeting agents in the treatment of patients with metastatic NSCLC. There exist other systematic reviews, which deal with the efficient treatment regimens for patients with metastatic NSCLC. However, they focus on later lines of treatment, do not include a quality assessment, or lack of current evidence [31, 32]. However, cost-effectiveness analyses aim at supporting the decision-making process regarding pricing and reimbursement of new technologies in health care systems.

Seven main results were derived:

1. The majority of studies indicate a cost-effectiveness of erlotinib compared to BSC in the first-line maintenance treatment of NSCLC. However, the results for subsequent treatment lines are ambiguous and do not allow firm conclusions to be drawn. (2) Three studies show a dominance of erlotinib when compared to docetaxel in subsequent-line settings. Thongprasert et al. [19], however, report no cost-effectiveness for patients with metastatic NSCLC. (3) The insights regarding bevacizumab in the first-line treatment of advanced non-squamous NSCLC are miscellaneous. Some studies indicate a potential cost-effectiveness [27, 28], whereas others report a very high ICER [15, 29, 30]. (4) The evidence indicates a possible cost-effectiveness of gefitinib, regardless of patients EGFR mutation status [19, 23]. Nevertheless, these results are based on only two studies. (5) Pharmacogenomic profiling is only considered by two studies [23, 26]. One study indicates that testing might not be cost-effective prior to the treatment with erlotinib [26] whereas another study shows that gene-guided gefitinib maintenance treatment is potentially cost-effective [23]. Hence, no final conclusion can be drawn. (6) No evidence was found for crizotinib and afatinib. There is a need for health economic analysis of the treatment with crizotinib and afatinib (7) The review discloses that the available evidence is of only fair quality; particularly when compared to other economic studies in the field of cancer [33]. However, only the results by Araújo et al. [17] appear to have been reported selectively to favor a medication.

Besides these main findings, the review shows a need for further research. Future studies should clarify the cost-effectiveness of erlotinib in subsequent treatment lines based on efficacy data from meta-analysis or medical reviews. Moreover, only one study exists that analyses the cost-effectiveness of the recently approved first-line treatment with erlotinib. The reliability of the results by Wang et al. [16], in addition, is limited. In terms of data validity, further cost-effectiveness analyses are also necessary for the treatment with bevacizumab. Additionally, only poor evidence exists for the treatment with gefitinib. Due to the EMA approval, further research should focus on the cost-effectiveness in all lines for the treatment of NSCLC with activating mutations of EGFR-tyrosine kinase [34]. Thereby, it is important to meet the international health economics standards when evaluations are conducted. Hence, the validity of the results is more reliable. Our quality assessment revealed that there are some criterions that are often not met. Particularly the plausible selection of data, the handling of uncertainty, the measurement of costs as well as the discussion of the direction and magnitude of potential biases were partly insufficient. Future research should take the standards more into account.

In addition, due to the rising importance of pharmacogenomic profiling in the pharmaceutical treatment of metastatic NSCLC, EGFR and ALK testing should also be considered in future models for the treatment with erlotinib and crizotinib, respectively. In general, the economic consequences are not to be foreseen. On the one hand, pharmacogenomic testing generates additional diagnostic costs for health care systems; on the other hand, it may reduce treatment costs due to a better response to these targeted agents. However, there is a lack of standard modeling techniques used in health economic evaluations for biomarker and diagnostic tests, as a recent review by Frank et al. [35] has shown in the case of metastatic colorectal cancer. Hence, further research should focus on clinical and economic evidence supporting pharmacogenomic profiling prior to the administration of targeted therapies in NSCLC.

There are limitations that need to be acknowledged regarding the present review. Systematic reviews are different from traditional narrative reviews or expert commentaries. They are transparent, rigorous and replicable [36]. However, other criteria that can influence policy making, including public opinion and expert advice, need to be considered. Systematic reviews do not equate meta-analysis but intend to reveal the best available evidence. Therefore, the literature search in the present review was conducted in many different databases and was updated shortly before the completion of this manuscript. In addition, it must be considered that there could be a publication bias which influences the available evidence. This problem may be particularly important here, since most studies are sponsored by stakeholders. Due to the ongoing research and development in the field of cancer the approval states are subject to frequent changes. Therefore the results of some studies included here are not valid for the present drug’s approval states.

Furthermore, the results of the quality assessment in this review should be considered with caution due to the subjective character. Two researches performed the assessment independently to minimize the subjectivity of the assessment. Afterwards the results were compared and discussed if differences in the assessment occurred. Additionally, the quality of the studies was assessed using the CHEC Instrument [37]. Both instruments are not directly comparable since the CHEC instrument does not involve the allocation of points. The trend of the assessment, however, was compared. No meaningful differences were apparent. However, there are quality aspects which have recently gained importance, e.g. if indirect comparison methods are used, and which the above mentioned instruments do not consider.

## Conclusion

All in all, this review underlines the high economic impact of targeted therapies in the treatment of NSCLC. A general conclusion regarding the cost-effectiveness of targeted therapies, however, cannot and should not be drawn, since the results depend on the concrete medication, the treatment line, the setting and the potential application of tests.

## Electronic supplementary material

Additional file 1: **Data extraction template.** (DOCX 12 KB)
