# Supplementary material for: A systematic review of the cost-effectiveness of targeted therapies for metastatic non-small cell lung cancer (NSCLC)
Source: BMC Pulm Med. 2014 Dec 4;14:192. doi: 10.1186/1471-2466-14-192 (PMC4269853; doi:10.1186/1471-2466-14-192)
Supplement: Supplementary file 1 — Additional file 1: Data extraction template. (DOCX 12 KB) [file 12890_2013_623_MOESM1_ESM.docx]

**Additional file 1: Data extraction template**

| # Nr. |
| --- |
| Title: |
| Authors: |
| Journal: |
| Year of publication |
| Study question |
| Population |
| Type of study / model |
| Primary outcome measure(s) |
| Interventions, comparator |
| Setting |
| Time horizon / cycle length |
| Measure of costs (sources and evaluation) |
| Measure of benefit (sources and evaluation) |
| Discounting |
| Perspective |
| Results |
| Sensitivity analysis |
| Authors' conclusions |
| Funding source  *Academic Institution   Healthcare Industry  Government   NGO  Public funds   Other* |
